# Differential completeness of spontaneous adverse event reports among hospitals/clinics, pharmacies, consumers, and pharmaceutical companies in South Korea

**DOI:** 10.1371/journal.pone.0212336

**Published:** 2019-02-14

**Authors:** In-Sun Oh, Yeon-Hee Baek, Hye-Jun Kim, Mose Lee, Ju-Young Shin

**Affiliations:** School of Pharmacy, Sungkyunkwan University, Suwon, Gyeonggi-do, South Korea; Jagiellonian University, POLAND

## Abstract

The differential pattern and characteristics of completeness in adverse event (AE) reports generated by hospitals/clinics, pharmacies, consumer and pharmaceutical companies remain unknown. Thus, we identified the characteristics of complete AE reports, compared with those of incomplete AE reports, using a completeness score. We used Korea Institute of Drug Safety and Risk Management-Korea Adverse Event Reporting System Database (KIDS-KD) between January 1, 2016 and December 31, 2016. The completeness score was determined out of a total of 100 points, based on the presence of information on temporal relationships, age and sex of patients, AE progress, name of reported medication, reporting group by profession, causality assessment, and informational text. AE reports were organized into four groups based on affiliation: hospitals/clinics, pharmacies, consumers, and pharmaceutical companies. Affiliations that had median completeness scores greater than 80 points were classified as ‘well-documented’ and these reports were further analyzed by logistic regression to estimate the adjusted odds ratios and 95% confidence intervals. We examined 228,848 individual reports and 735,745 drug-AE combinations. The median values of the completeness scores were the highest for hospitals/clinics (95 points), followed by those for consumers (85), pharmacies (75), and manufacturers (72). Reports with causality assessment of ‘certain’, ‘probable’, or ‘possible’ were more likely to be ‘well-documented’ than reports that had causality assessments of ‘unlikely’. Serious reports of AEs were positively associated with ‘well-documented’ reports and negatively associated with hospitals/clinics.

## Introduction

Although the spontaneous reporting system is a keystone of pharmacovigilance, the incompleteness of data is a major concern for causality assessment [1, 2]. Since causal assessment of adverse events (AEs) cannot be made without demographic or clinical information, completeness in reporting is important for the early detection of adverse drug reactions. Identifying the factors associated with reporting completeness is critical for enhancing quality management systems—an essential component of good pharmacovigilance practices [3, 4]. Higher reporting completeness is associated with the seriousness of an AE [5].

Including different reporting groups and using an integrated interpretation are important in evaluating completeness, as the reported AE will vary with the reporting group. Reports from community pharmacists are important owing to their wide distribution and geographical accessibility [6]. Although reports from pharmacists had lower completeness scores than reports from physicians, significant differences in the distribution of AEs were observed [7]. In addition, consumer reports have the potential benefit of detecting unknown signals because they provide different categories of AEs for different types of medicines, unlike reports by healthcare professionals [8, 9].

Few studies have used a large spontaneous reporting database to evaluate the characteristics of completeness by reporting group. Given the scarcity of information on the factors associated with completeness of AE reports, established reporting systems can be leveraged to describe and compare the characteristics of the different reporting groups. Hence, we compared the differences in completeness by reporting group and categorized the determinants by following four affiliations: hospitals/clinics, pharmacies, pharmaceutical companies, and consumers.

## Materials and methods

This study was approved by the Sungkyunkwan University’s Institutional Review Board(SKKU-IRB-2017-09-007), which waived the requirement for informed consent.

### The Korea Adverse Event Reporting System (KAERS) database

The Korea Adverse Event Reporting System was set up in 1988, and the reporting rate increased rapidly following the designation of three university hospitals as Korean Regional Pharmacovigilance Centers in 2006, and the establishment of the Korea Institute of Drug Safety and Risk Management-Korea Adverse Event Reporting System Database (KIDS-KD) in 2012 [10]. The KAERS database was developed by KIDS in 2012 to manage AE reports effectively. This computerized AE reporting system includes voluntary reporting by healthcare workers and the general public, as well as mandatory reporting by manufacturers for serious and unexpected events, using a standardized form [11]. Reports from the Korean Regional Pharmacovigilance Centers accounted for 71.5% (163,676 reports) of the KAERS database, and among these, hospitals/clinics accounted for the majority.

This database includes information on AE codes, serious AEs, suspected drug information, reporting group by profession, reporting group by affiliation, and causality assessment information. In the database, the suspect drugs are arranged in accordance with the Anatomical Therapeutic Chemical (ATC) classification system. AEs are described using the preferred terms (PTs) recommended by the World Health Organization Adverse Reaction Terminology (WHO-ART). In the future, these will be changed to terms in the Medical Dictionary for Regulatory Activities (MedDRA) according to the recommendation of the WHO [12, 13]. A serious AE corresponds to any AE resulting in a death, a life-threatening situation, an inpatient hospitalization or prolongation of existing hospitalization, a persistent or significant disability, congenital abnormality or birth defect, or other medically important conditions as defined by KIDS.

### Selection of AE reports

We accessed all AE reports that were filed in the KAERS database between January 1, 2016 and December 31, 2016, and categorized the reporting group by affiliation where this information was available. Since each report consisted of combinations of drugs defined according to the ATC classification system and AEs classified according to WHO-ART, we included individual reports that comprised of combinations of drug-AEs. We then conducted an analysis based on the reporting group by affiliation.

### Reporting group by affiliation and reporting group by profession

We used four different reporting groups by affiliation: hospitals/clinics, pharmacies, manufacturers, and consumers. Reporting groups were defined as follows: hospitals/clinics, if they were either medical institutions or health centers; pharmacies, if pharmacy reports were reported to the Korean Pharmaceutical Association; manufacturers if they were reported by pharmaceutical companies; and consumers, for those that were not reported through the aforementioned affiliations.

Reporting groups by profession consisted of doctors, pharmacists, nurses, and consumers. These categories were based on information provided about the occupation of the person who made the initial report on the AEs.

### Characteristics of AE reports

For this study, we analyzed the frequency and proportion of patient age, sex, reporting year, date of prevalence, and reporting group by profession among hospitals/clinics, pharmacies, manufacturers, and consumers. Age consisted of six subgroups: neonates aged <28 days; infants aged 28 days to < 2 years; children aged 2 to < 12 years; adolescents aged 12 to < 19 years; adults aged 19 to < 65 years; and the elderly aged ≥65 years. In order to ensure comparability, the age subgroups were merged into three groups (<19, 19–65, and >65). For each report, if information was missing in age, sex, month, or reporting group by profession, it was classified as ‘No information’ for that particular variable.

Based on the International Conference on Harmonization (ICH) E2D Guidelines, we also classified all adverse reactions into ‘serious reports of AEs’ or ‘not serious reports of AEs’. Causality assessment was performed using the six categories classified by the World Health Organization-Uppsala Monitoring Center (WHO-UMC) criteria: certain, probable, possible, unlikely, unclassified, and unassessable [14]. This causality was primarily estimated by the adverse drug reaction monitoring team at each Regional Pharmacovigilance Center and was subsequently re-estimated and validated by KIDS healthcare professionals [15]. In this study, our research interest was not in the level of causality assessment but in the presence of causality; therefore, we merged causality information into three groups: presence of causality (certain, probable, or possible), absence of causality (unlikely), and other (unclassified or unassessable).

### Completeness score for hospitals/clinics, pharmacies, manufacturers, and consumers

The completeness score comprised a total of 100 points. From the 100 points, 20 points were accorded to temporal relationships, 15 points for information on the patient, 5 points for progress of AEs, 30 points for information on prescription of medicines, 5 points for information on reporting group by profession, 5 points for causality assessment, and 20 points for informational text (S1 Appendix).

Based on information provided in the AE reports, reports were classified into two groups: ‘well-documented’ and ‘poorly documented.’ A full completeness score was given only when there were no missing values among all criteria of the completeness score assessment. We used the median score to determine the criteria for classifying reports as ‘well-documented’ or ‘poorly documented.’ Completeness scores greater than the overall median score were considered ‘well-documented’; scores below the overall median score were considered ‘poorly documented.’

### Statistical analysis

The demographic and clinical characteristics of study participants were analyzed using medians, means, and standard deviations (SD) for the continuous variables. Frequencies and percentages were used to analyze the categorical variables. The chi-square test was used to compare categorical variables, and p-values < 0.05 were considered to be statistically significant. To identify the characteristics of a ‘well-documented’ AE report, we conducted a multivariate logistic regression analysis with ‘well-documented’ AE report as the dependent variable, and estimated the adjusted odds ratios (aORs) and 95% confidence interval (CI). Our multivariate logistic regression model included sex, age, serious AEs, and causality assessment as independent variables.

We also carried out a subgroup analysis for each affiliation to determine the influence of the reporting group’s profession. We conducted a multivariate logistic regression model to reveal the association between the completeness score and the sex, age, serious AEs, and causality assessment for each profession (doctors, pharmacists, nurses, and consumers). We determined if there were differences between characteristics associated with high completeness scores for each profession nested within each affiliation. We used the SAS 9.4 software (SAS Institute Inc., Cary, NC, USA) for all statistical analyses.

## Results

**Fig 1:**
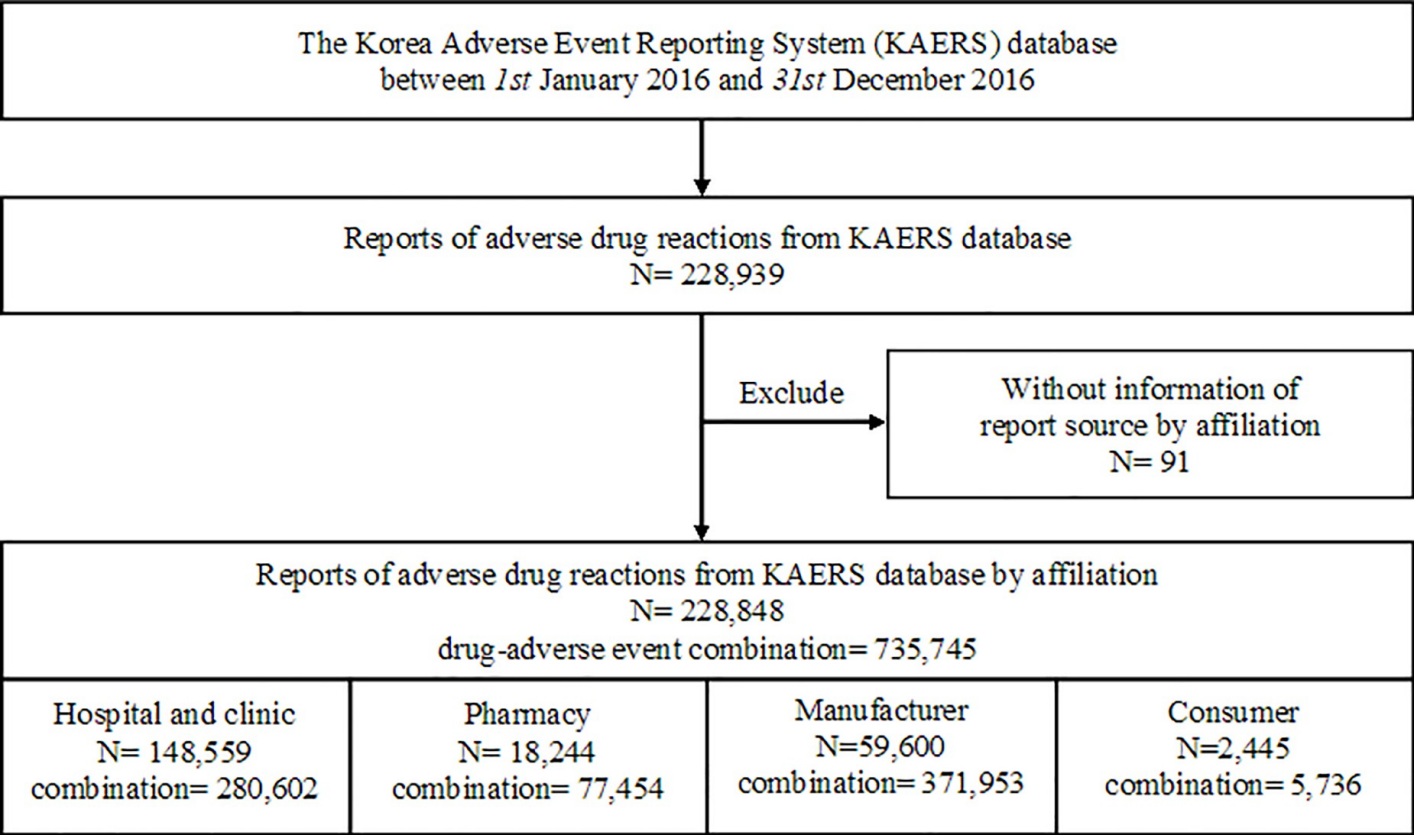
Study flow diagram describing reports of adverse drug reactions and their combinations.

Of the 228,939 individual reports collected in the KAERS database, 91 were excluded because of a lack of information about reporting group by affiliation (Fig 1). A total of 228,848 individual reports and 735,745 drug-AE combinations were included for analysis. About 64.9% of the reports were from hospitals/clinics (N = 148,559; combination = 280,602), 8.0% were from pharmacies (N = 18,244; combination = 77,454), 26.0% were from manufacturers (N = 59,600; combination = 371,953), and 1.1% were from consumers (N = 2,445; combination = 5,736).

We describe characteristics of the reporting groups by affiliation according to age, sex, month, and reporting group by profession, with all chi-square test results being statistically significant (Table 1). When classified by age, adults (19–65 years) comprised the largest proportion of reports for all affiliations, which was also the case for overall reports. For all variables except reporting group by profession, the proportion of criteria that had ‘no information’ was largest from manufacturers. When classified by reporting groups by profession, more than 90% were pharmacists and consumers for the affiliations of pharmacy and consumers, respectively. Nurses and doctors were the major professions when the affiliations were hospitals/clinics and manufacturer, respectively. Furthermore, from a total of 107,694 reports made by nurses, 106,494 were from hospitals/clinics, whereas, for doctors and consumers, the vast majority of the reports were made to manufacturers.

**Table 1:** Characteristics of the reporting groups by affiliation from January 2016 to December 2016.

	Total (N = 228,848)		Hospitals/ clinics (N = 148,559)		Pharmacies (N = 18,244)		Manufacturers (N = 59,600)		Consumers (N = 2,445)		*p-value*
	N	%	N	%	N	%	N	%	N	%	
**Age**^a^											< 0.001
Neonates	95	0.0	28	0.0	0	0.0	67	0.1	0	0.0	
Infants	2,787	1.2	1,108	0.8	723	4.0	838	1.4	118	4.8	
Children	4,153	1.8	3,054	2.1	366	2.0	671	1.1	62	2.5	
Adolescents	4,338	1.9	3,434	2.3	382	2.1	490	0.8	32	1.3	
Adults	127,957	55.9	91,681	61.7	11,673	64.0	22,722	38.1	1,881	76.9	
Elderly	64,578	28.2	45,515	30.6	4,981	27.3	13,912	23.3	170	7.0	
No information	24,940	10.9	3,739	2.5	119	0.7	20,900	35.1	182	7.4	
**Sex**											< 0.001
Male	91,136	39.8	61,713	41.5	5,437	29.8	23,096	38.8	890	36.4	
Female	130,631	57.1	86,069	57.9	12,224	67.0	30,794	51.7	1,544	63.2	
No information	7,081	3.1	777	0.5	583	3.2	5,710	9.6	11	0.5	
**Month**											< 0.001
January	17,322	7.6	11,660	7.9	1,164	6.4	4,290	7.2	208	8.5	
February	15,583	6.8	9,888	6.7	1,009	5.5	4,413	7.4	273	11.2	
March	16,615	7.3	11,302	7.6	1,375	7.5	3,714	6.2	224	9.2	
April	16,927	7.4	11,134	7.5	1,573	8.6	4,013	6.7	207	8.5	
May	19,608	8.6	11,718	7.9	1,472	8.1	6,089	10.2	329	13.5	
June	18,842	8.2	13,194	8.9	1,301	7.1	4,093	6.9	254	10.4	
July	20,159	8.8	13,241	8.9	1,402	7.7	5,377	9.0	139	5.7	
August	19,189	8.4	12,856	8.7	1,445	7.9	4,792	8.0	96	3.9	
September	17,453	7.6	11,343	7.6	1,378	7.6	4,626	7.8	106	4.3	
October	17,604	7.7	12,090	8.1	1,707	9.4	3,679	6.1	128	5.2	
November	19,412	8.5	11,666	7.9	1,778	9.8	5,752	9.7	216	8.8	
December	17,543	7.7	11,829	8.0	2,480	13.6	2,990	5.0	244	10.0	
No information	12,591	5.5	6,638	4.5	160	0.9	5,772	9.7	21	0.9	
**Reporting group by profession**											< 0.001
Doctor	56,719	24.8	22,867	15.4	1	0.0	33,849	56.8	2	0.1	
Pharmacist	30,834	13.5	11,122	7.5	18,115	99.3	1,596	2.7	1	0.0	
Nurse	107,964	47.2	106,494	71.7	2	0.0	1,468	2.5	0	0.0	
Consumer	22,413	9.8	2,177	1.5	50	0.3	17,964	30.1	2,222	90.9	
No information	10,918	4.8	5,899	4.0	76	0.4	4,723	7.9	220	9.0	

a. Neonates (aged <28 days); Infants (aged 28 days<24 months); Children (aged 24 months<12 years); Adolescents (aged 12<19 years); Adults (aged 19 years<65 years); Elderly (aged ≥65 years)

Table 2 describes the characteristics of ‘serious’ and ‘not serious’ reports of AEs and causality assessments according to the reporting group by affiliation. The overall proportion of serious AEs was 9.7%. For manufacturers, the proportion of serious AEs was higher than those of other reporting groups by affiliation (26.3%). ‘No information’ was zero for all reporting groups by affiliation. By affiliation, manufacturers had the highest proportion of reports for which causality was "unlikely", while the highest proportion of "no information" was among consumers and manufacturers.

**Table 2:** Distribution of serious adverse events and causality assessments among reporting groups by affiliation from January 1, 2016 to December 31, 2016.

	Total		Hospitals/ clinics		Pharmacies		Pharmaceutical companies		Consumers		*p-value*
	N	%	N	%	N	%	N	%	N	%	
**Serious** **adverse events**^a^											<0.001
Yes	22,197	9.7	6,318	4.3	52	0.3	15,695	26.3	132	5.4	
No	206,651	90.3	142,241	95.7	18,192	99.7	43,905	73.7	2,313	94.6	
**Causality assessment**											<0.001
Certain	6,506	2.8	5,211	3.5	354	1.9	941	1.6	0	0.0	
Probable	56,777	24.8	54,064	36.4	1,286	7.0	1,426	2.4	1	0.0	
Possible	103,432	45.2	80,711	54.3	13,053	71.5	9,467	15.9	201	8.2	
Unlikely	19,774	8.6	2,250	1.5	3,494	19.2	14,020	23.5	10	0.4	
Unclassified	2,862	1.3	970	0.7	16	0.1	1,872	3.1	4	0.2	
Unassessable	4,371	1.9	251	0.2	0	0.0	4,119	6.9	1	0.0	
Not applicable	116	0.1	6	0.0	0	0.0	110	0.2	0	0.0	
No information	35,010	15.3	5,096	3.4	41	0.2	27,645	46.4	2,228	91.1	

a. Since it is mandatory to respond to any serious AEs regardless of the reporter, there was no missing information on serious adverse events.

Table 3 shows the distribution of completeness scores by affiliation. Median values of completeness scores were the highest for hospitals/clinics (95 points), followed by those of consumers (85 points), pharmacies (75 points), and manufacturers (72 points). Hospitals had the highest proportion (93.6%) in the group of completeness scores greater than the overall median, greatly exceeding all other affiliations, with the mean and median results showing similar results as the distribution. In addition, hospitals had a much higher proportion than any other affiliation of ‘well-documented’ reports that had a completeness score greater than the median value, whereas pharmacies and consumers had a very low proportion of ‘well-documented’ reports.

**Table 3:** Distribution of completeness score, mean, median, and completeness score for each reporting group by affiliation.

	Total		Hospitals/ clinics		Pharmacies		Manufacturers		Consumers		*p-value*
	N	%	N	%	N	%	N	%	N	%	
**Distribution of completeness score (N, %)**											<0.001
<20 points	1	0	1	0	0	0	0	0	0	0	
20 points–40 points	3,445	1.5	819	0.6	35	0.2	2,588	4.3	3	0.1	
40 points–60 points	16,192	7.1	2,661	1.8	176	1	13,176	22.1	179	7.3	
60 points–80 points	39,307	17.2	6,098	4.1	11,521	63.1	21,270	35.7	418	17.1	
≥80 points	169,912	74.2	138,980	93.6	6,521	35.7	22,566	37.9	1,845	75.5	
**Total cases (N, %)**	228,848	100	148,559	100	18,244	100	59,600	100	2,445	100	<0.001
Mean (standard deviation)	85.6(16.5)		93.6(10.3)		73.4(7.8)		69.7 (17.2)		78.9 (10.0)		
Median (Q1–Q3)	95.5 (78.3–96.1)		95.0 (95.0–100.0)		75.0 (65.0–80.0)		72.0 (55.0–83.3)		85.0 (80.0–85.0)		
**Completeness score**^a^ ^b^ ^c^**(N, %)**											<0.001
Well-documented (above median)	123,142	53.8	118,996	80.1	1	0.0	4,145	7.0	0	0.0	
Poorly documented (below median)	105,706	46.2	29,563	19.9	18,243	100	55,455	93.0	2,445	100	

a. The general characteristics of the patients are calculated after excluding missing values (N = 91) from the reporting group by affiliation.

b. As the distribution of scores was skewed to the left, we used the median score to determine the criteria for categorizing the completeness score.

c. Kolmogorov-Smirnov test results used a median instead of an average to test the assumption of normality.

Table 4 presents the odds ratios for high completeness scores associated with reporting group by affiliation. Among 735,745 combinations of reports, pharmacies and consumers had too few ‘well-documented’ reports; therefore, we could not estimate the odds ratios for pharmacies and consumers. The aOR for hospitals/clinics associated with serious AEs was 0.71 (95% CI: 0.68–0.73), whereas that of manufacturers was 1.34 (95% CI: 1.31–1.38). However, the aORs associated with the presence of causality for hospitals/clinics and manufacturers were 2.41 (95% CI: 2.26–2.56) and 0.78 (95% CI: 0.74–0.83), respectively.

**Table 4:** Logistic regression for the factors associated with completeness score of reporting groups by affiliation according to sex, age, serious adverse event, and causality assessment.

	Total (N = 735,745)		Hospitals/clinics (N = 280,602)		Manufacturers (N = 371,953)	
	aOR	95% CI	aOR	95% CI	aOR	95% CI
**Sex**						
Male	Reference		Reference		Reference	
Female	0.79	(0.79–0.80)	0.87	(0.86–0.89)	1.02	(0.99–1.04)
**Age**						
≥65 years	Reference		Reference		Reference	
19 years–65 years	1.04	(1.02–1.05)	0.95	(0.93–0.97)	1.33	(1.29–1.36)
0 days–19 years	0.80	(0.77–0.82)	1.03	(0.98–1.08)	0.32	(0.27–0.36)
**Serious adverse event**						
No	Reference		Reference		Reference	
Yes	1.09	(1.07–1.11)	0.71	(0.68–0.73)	1.34	(1.31–1.38)
**Causality assessment**^a^						
Unlikely	Reference		Reference		Reference	
Certain, Probable, Possible	12.35	(12.02–12.68)	2.41	(2.26–2.56)	0.78	(0.74–0.83)

a. 'Unclassified', 'Unassessable', and 'Not applicable' are not shown.

In the subgroup analyses of hospitals/clinics, for all professions, females had significantly decreased odds ratios of association with high completeness scores, as compared with those of males. Doctors were observed to have an increased association with completeness for serious AEs, whereas nurses showed opposite results. Moreover, for all professions, if the causality assessment was not ‘unlikely,’ the completeness scores were high (Table 5).

**Table 5:** Subgroup analysis for difference of characteristics associated with completeness scores by profession, restricted to reports from hospitals/clinics.

	Doctors (N = 53,541)		Pharmacists (N = 23,074)		Nurses (N = 190,667)		Consumers (N = 4,725)	
	aOR	95% CI	aOR	95% CI	aOR	95% CI	aOR	95% CI
Sex								
Male	Reference		Reference		Reference		Reference	
Female	0.77	(0.74–0.80)	0.72	(0.68–0.77)	0.94	(0.92–0.97)	0.76	(0.67–0.86)
Age								
≥65 years	Reference		Reference		Reference		Reference	
19 years–65 years	0.80	(0.77–0.84)	1.18	(1.10–1.26)	0.95	(0.92–0.97)	0.94	(0.81–1.09)
0 days–19 years	0.65	(0.59–0.72)	1.16	(1.00–1.37)	1.11	(1.04–1.18)	3.10	(1.43–6.74)
Serious adverse event								
No	Reference		Reference		Reference		Reference	
Yes	1.39	(1.31–1.48)	0.93	(0.83–1.05)	0.51	(0.48–0.54)	1.05	(0.72–1.55)
Causality assessment^a^								
Unlikely	Reference		Reference		Reference		Reference	
Certain, Probable, Possible	3.43	(3.01–3.91)	2.46	(2.16–2.80)	1.39	(1.26–1.54)	4.08	(2.56–6.50)

a. 'Unclassified', 'Unassessable', and 'Not applicable' are not shown.

In the subgroup analysis of manufacturers, doctors and nurses, unlike consumers, had significant;y higher completeness scores for serious AEs. When causality was confirmed, nurses and consumers had large ORs, inferring a high completeness score, in contrast to those of doctors (Table 6).

**Table 6:** Subgroup analysis for differences in characteristics associated with completeness scores by profession, restricted to reports from manufacturers.

	Doctors (N = 291,461)		Pharmacists^b^ (N = 2,539)		Nurses (N = 2,713)		Consumers (N = 59,229)	
	aOR	95% CI	aOR	95% CI	aOR	95% CI	aOR	95% CI
Sex								
Male	Reference		Reference		Reference		Reference	
Female	1.13	(1.10–1.16)	-		1.20	(0.76–1.88)	0.75	(0.69–0.81)
Age								
≥65 years	Reference		Reference		Reference		Reference	
19–65 years	1.57	(1.52–1.61)	-		1.16	(0.56–2.38)	0.75	(0.69–0.81)
0 days–19 years	0.20	(0.17–0.24)	-		2.28	(0.71–7.38)	1.01	(0.80–1.28)
Serious adverse event								
No	Reference		Reference		Reference		Reference	
Yes	1.56	(1.52–1.60)	-		2.43	(1.36–4.32)	0.63	(0.59–0.69)
Causality assessment^a^								
Unlikely	Reference		Reference		Reference		Reference	
Certain, Probable, Possible	0.50	(0.46–0.54)	-		4.94	(2.99–8.16)	1.96	(1.76–2.18)

a. ‘Unclassified,’ ‘Unassessable,’ and ‘Not applicable’ are not shown.

b. The aOR and 95% CI for pharmacists were not estimated owing to skewed data.

## Discussion

The omission of key information in AE reports has been a critical hurdle for the quality management of signal detection [2, 5, 16, 17]. Our study was designed to identify factors associated with completeness in AE reports. It is notable that reports that include causality assessment had 12.4 increased odds of being “well-documented”, when compared with reports where causality was assessed as “unlikely”. In addition, hospital and clinics had higher median completeness scores than those of pharmacies. Sixty-five percent of reports were collected from hospital and clinics. Nurse reports made up the highest proportion (55%) of reports from all professions, a marked difference from a previous finding in which nurse reports comprised 6.5% of all AE reports [18]. Our results demonstrated the determinants of greater data completion in different report groups by using a nationwide AE reporting system.

We found that 99.4% of pharmacy reports were poorly documented, whereas 80.9% of reports from hospitals/clinics were well-documented. Since community pharmacies are based on ambulatory settings and have less access to clinical information, including indications, only 0.1% of pharmacy reports had information on indications, which led to lower completeness in their reports. The Korean Pharmaceutical Association, which supplied the only group of AE reports from community pharmacies, was designated as a regional pharmacovigilance center in 2013 [6, 19]. Although issues regarding the quality of the reports have been raised, the association has generated the highest number of reports among 27 regional pharmacovigilance centers [20]. In contrast, hospital and clinics were more likely to have access to the demographic and clinical information of patients, resulting in high completeness.

Contrary to a previous finding reporting an association between seriousness of event and increased completeness [5], our logistic regression results were statistically insignificant. Moreover, an inverse association between the two was observed for hospitals/clinics. However, significant results were observed in the hospital and clinic subgroup analyses. Nurses had a lower chance of submitting ‘well-documented’ reports for serious AEs than non-serious AEs. This can be explained in the context that nurses are involved with medication errors in the process of administering and dispensing medications [21, 22] in nursing practice. Medication errors—preventable mistakes in prescribing or delivering medication to patients [23]—are associated with adverse drug reactions [24]. The psychological burden caused by the possible risk of legal responsibility for serious AEs might lead to intentional omissions in reporting serious AEs. Conversely, doctors had 1.3-fold increased odds of ‘well-documented’ reports for serious AEs.

In the logistic regression analyses, ORs were not estimated for pharmacies and consumers because of their highly skewed completeness scores. In the S2 Appendix, we explore the proportion of each completeness evaluation criterion that had ‘no information,’ which was included in the calculation of the completeness score. The proportion of ‘no information’ for the criterion ‘indication’ in reports filed by pharmacies and consumers was greater than 99.7% (S2 Appendix). Since this particular criterion accounts for 10 points of the total score, the completeness scores of pharmacies and consumers could not have exceeded the overall median of 95 points.

The low completeness score of manufacturer reports corroborates a study of FDA reports that also observed poor completeness of reports by pharmaceutical companies for serious adverse drug events [25]. In the manufacturer subgroup analyses, heterogeneity of the results was observed when data were classified by reporting profession. Consumer reports showed an inverse association between seriousness of AEs and completeness, unlike those from healthcare specialists, including doctors, pharmacists, and nurses. Notably, doctors were more likely to submit ‘poorly documented’ reports for cases suspected to have causal association when reporting to manufacturers. This result is discordant with the results from the hospitals/clinics subgroup analysis, in which doctors had 3.43-fold increased odds of submitting ‘well-documented’ reports that are suspected to have causal association. When reporting to manufacturers, doctors may be less obligated to complete reports. However, since AE reporting is used for performance evaluation in hospitals designated as regional pharmacovigilance centers, this may have resulted in a higher motivation for doctors to complete the AE reports [10]. Therefore, affiliatory and psychological motivations might account for the inconsistency in the magnitude of association between main and subgroup analyses of AE reports.

Our study has several strengths. By using the nationwide AEs reporting database, we generated representative results because of the inclusion of all reports from different reporting groups in South Korea. To the best of our knowledge, this is the first study identifying determinants of completeness in AE reports in South Korea. Our results have particular importance in that we used reporting groups across different affiliations and professions of reporters with distinct features [18, 26, 27]. In addition, we identified the current status of completeness in AE reporting systems using recent data.

However, our study is subject to the following limitations. First, reports from consumers and pharmacies were excluded from the logistic regression model owing to the selection of reports with scores above the overall median completeness score. Since hospitals/clinics reported the majority of such reports, the distribution of completeness scores is skewed negatively by reports from this affiliation. Our findings of characteristics associated with data completeness are restricted to hospitals/clinics, and manufacturers, and therefore, may not apply to other affiliations (consumers and pharmacies). More generalizable results could have been achieved by including more reports from consumers and pharmacies using a weighted method. Second, spontaneous reports are based on the reporters’ subjective judgements. Thus, concerns about objectivity and quality stability may arise. However, this is not a significant hurdle for our objective, as we are interested in identifying the determinants of completeness, with a focus on variables with missing values. Third, our study is based on a cross-sectional design, so no causal interpretation can be made. Moreover, the findings in the main and subgroups analyses are inconsistent. Although we interpreted the results based on previous findings and affiliatory contexts, the possibility of misinterpretation exists. Finally, there is a possibility of duplication in reports, as the KAERS database is anonymized. Such duplication could occur if multiple reports are made for the same event.

From 228,848 individual AE reports in the database, we found the differential completeness score for reporting source by affiliation to be highest for hospitals and clinics, followed by those of consumers, pharmacies, and manufacturers. Reports suspected to have causal association in the causality assessment were more likely to have better completeness in all reports by affiliation, while seriousness of AEs was not associated with completeness of reports by hospitals/clinics. Low completeness in reports by pharmacies and manufacturers could be due to the difficulty of collecting information on the details of AEs such as indication, medical history or inability to follow-up with patients after the onset of AEs. Given that pharmacists and manufacturers are equally important stakeholders of AEs as well as healthcare professionals, systematic training programs such as developing standardized coding guidelines should be provided in adverse reports to enhance completeness of reports from these affiliations. Implementation of strategies to engage healthcare professionals will be critical for enhancing completeness in spontaneous reports and early signal detection.

## Supporting information

S1 Appendix Standard of completeness scores in the Korea Adverse Event Reporting System (KAERS) database. (DOCX)

S2 Appendix The number of reports having missing values in each category of the adverse event reporting form, used to calculate completeness scores, by reporting group by profession. (DOCX)
