# Supplementary material for: Differential completeness of spontaneous adverse event reports among hospitals/clinics, pharmacies, consumers, and pharmaceutical companies in South Korea
Source: PLoS One. 2019 Feb 14;14(2):e0212336. doi: 10.1371/journal.pone.0212336 (PMC6375612; doi:10.1371/journal.pone.0212336)
Supplement: S1 Appendix — (DOCX) [file pone.0212336.s001.docx]

**S1 Appendix.** Standard of completeness scores in the Korea Adverse Event Reporting System (KAERS) database.

| Classification | Details | Score |
| --- | --- | --- |
|  |  |  |
| Temporal relationship | Adverse event symptom occurrence date | 10 |
|  | Start date of administration | 10 |
| Information on patient | Sex | 5 |
|  | Age | 5 |
|  | Patient disease history/ drug usage history | 5 |
| Progress of adverse events | Progress of adverse events | 5 |
| Information on prescription of medicines | One-time dose | 5 |
|  | Number of doses (frequency of administration) | 5 |
|  | Measures on medicines etc. | 5 |
|  | Adverse event after re-administration | 5 |
|  | Indication | 10 |
| Information on report group by profession | Information on reporting group by profession | 5 |
| Causality assessment | Causality assessment | 5 |
| Informational text | Details of disease history etc. | 5 |
|  | Adverse event or inspection details | 5 |
|  | Comprehensive opinion | 10 |
| **Completeness score** | | **100** |
|  |  |  |
