# Supplementary material for: Differential completeness of spontaneous adverse event reports among hospitals/clinics, pharmacies, consumers, and pharmaceutical companies in South Korea
Source: PLoS One. 2019 Feb 14;14(2):e0212336. doi: 10.1371/journal.pone.0212336 (PMC6375612; doi:10.1371/journal.pone.0212336)
Supplement: S2 Appendix — (DOCX) [file pone.0212336.s002.docx]

**S2 Appendix.** The number of reports having missing values in each category of the adverse event reporting form, used to calculate completeness scores, by reporting group by profession.

|  | **Hospitals and clinics (N = 280,602)** | | **Pharmacies (N = 77,454)** | | **Manufacturers (N = 371,953)** | | **Consumers (N = 5,736)** | |
| --- | --- | --- | --- | --- | --- | --- | --- | --- |
|  | N | % | N | % | N | % | N | % |
|  |  |  |  |  |  |  |  |  |
| Sex | 1,749 | 0.6 | 1,528 | 2.0 | 11,518 | 3.1 | 17 | 0.3 |
| Age | 7,984 | 2.8 | 151 | 0.2 | 67,664 | 18.2 | 283 | 4.9 |
| Indication of use | 19,202 | 6.8 | 77,377 | 99.9 | 86,124 | 23.2 | 5,719 | 99.7 |
| Dosage | 17,022 | 6.1 | 1,522 | 2.0 | 140,934 | 37.9 | 2,286 | 39.9 |
| Number of dose | 11,792 | 4.2 | 19 | 0.0 | 226,113 | 60.8 | 1,073 | 18.7 |
| Onset date of treatment | 9,801 | 3.5 | 223 | 0.3 | 58,947 | 15.8 | 523 | 9.1 |
| Measures taken to treat the adverse reaction | 9,276 | 3.3 | 23,165 | 29.9 | 138,979 | 37.4 | 241 | 4.2 |
| Result of re-challenge | 161,580 | 57.6 | 43,557 | 56.2 | 188,873 | 50.8 | 1,080 | 18.8 |
| Date of onset | 9,932 | 3.5 | 202 | 0.3 | 54,016 | 14.5 | 374 | 6.5 |
| Progress of adverse event | 7,348 | 2.6 | 36,129 | 46.6 | 18,956 | 5.1 | 169 | 2.9 |
| Information of primary reporter | 2,044 | 0.7 | 81 | 0.1 | 9,932 | 2.7 | 1 | 0.0 |
| Medical history | 67,296 | 24.0 | 23,023 | 29.7 | 145,329 | 39.1 | 5,690 | 99.2 |
|  |  |  |  |  |  |  |  |  |
